# Effect of Tungsten Nanolayer Coating on Si Electrode in Lithium-ion Battery

**DOI:** 10.1186/s11671-018-2460-2

**Published:** 2018-02-21

**Authors:** Byung Dae Son, Jun Kyu Lee, Woo Young Yoon

**Affiliations:** Department of Materials Science and Engineering, Korea University, Anam-dong, Seongbuk-gu, Seoul, 136-713 Republic of Korea

**Keywords:** Lithium-ion battery, Silicon anode, Electrochemical reaction, Physical vaporization deposition

## Abstract

Tungsten (W) was coated onto a silicon (Si) anode at the nanoscale via the physical vaporization deposition method (PVD) to enhance its electrochemical properties. The characteristics of the electrode were identified by scanning electron microscopy (SEM), transmission electron microscopy (TEM), energy dispersive X-ray analysis, and electron probe X-ray microanalysis. With the electrochemical property analysis, the first charge capacities of the W-coated and uncoated electrode cells were 2558 mAh g^− 1^ and 1912 mAh g^− 1^, respectively. By the 50th cycle, the capacity ratios were 61.1 and 25.5%, respectively. Morphology changes in the W-coated Si anode during cycling were observed using SEM and TEM, and electrochemical characteristics were examined through impedance analysis. Owing to its conductivity and mechanical properties from the atomic W layer coating through PVD, the electrode improved its cyclability and preserved its structure from volumetric demolition.

## Background

Silicon (Si) is one of the most attractive energy source elements that can be used as an anode because of its high-specific capacity (4200 mAh g^− 1^), which is 10 times higher than that of graphite [1]. However, Si experiences problematic volumetric expansion during charging and discharging processes, and the expansion causes a 300% change in lattice volume [2–5]. This results in cracking and disintegration of the electrode, leading to active material loss, a decrease in electrical contact, and eventual degradation of electrical properties. Additionally, the low electrical conductivity of Si is a barrier to its use as an electrode material.

Therefore, methods for improving the electrochemical properties of Si electrodes are of high interest, and extensive research has been conducted to solve the problems associated with the Si electrode, such as using electrodes with a carbon (C) composite composition, multidimensional structures, and metal-alloyed forms [6–12]. In particular, for active material methods used in shockproofing, many studies have pursued approaches for coating the subject with various materials [13–16]. Conductive materials such as carbon, metal alloys, and even conductive polymers have been employed to restrain the expansion effect, and they have provided not only a buffering effect but also charge transportation enhancement. However, these research methods have limitations regarding their use in commercial applications because of their detailed fabrication procedures.

Physical vaporization deposition (PVD) produces a uniform coating on a substrate at the nanometer to visible scale through the process of atomic deposition [17–20]. This versatile technique can be applied in various fields to enable the deposition of every inorganic material type and even some organic materials. Additionally, because this method induces less resistance than chemical deposition with a tight layer formed by heterogeneous nucleation and growth [21], mechanical properties such as wear resistance and hardness are improved greatly.

In this study, a Si electrode was coated with tungsten (W) using the PVD method to provide a buffer layer and increase its conductivity. Among all metals in pure form, W has the highest tensile strength and superior hardness [22, 23]. In addition, Hornik et al. [24] studied the effect of W PVD by magnetron sputtering on ceramic substrates and showed that the W coating can function suitably for substrates with low hardness or wear resistance. By applying a W nanolayer to the electrode surface, the electrochemical properties and morphologies of the Si electrode were examined using various analytical techniques. This W nanolayer application showed improved electrochemical properties and sustained structural safety.

## Experimental

### Fabrication of Electrodes

**Fig. 1:**
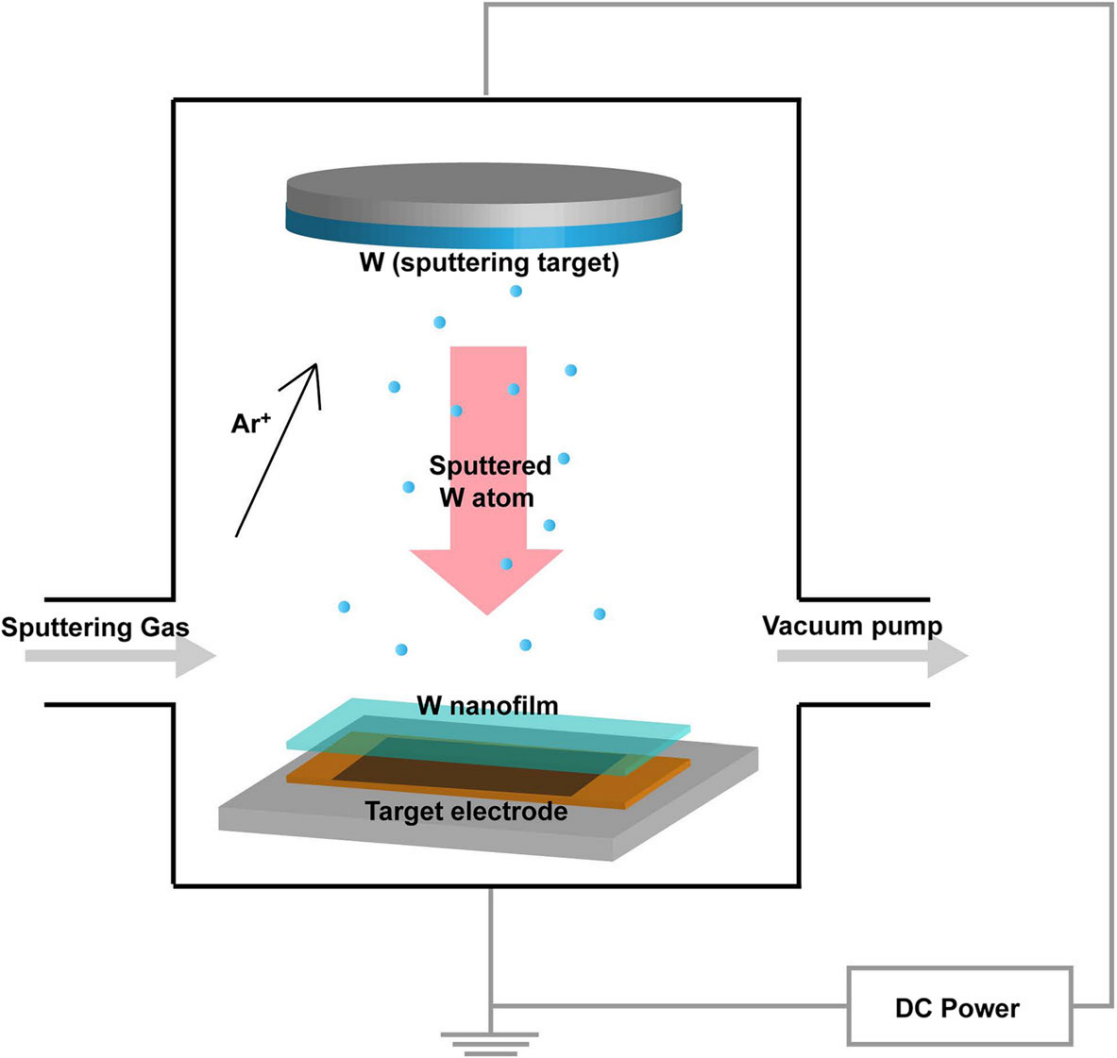
Schematic of physical vapor deposition for W coating

Si electrodes were fabricated using a casting method with 40 wt% Si nanopowder (≤ 100 nm), 40 wt% Denka Black as a conductive material, and carboxymethyl cellulose as a binder. These substances were dissolved in deionized water to form a slurry. The slurry was then coated onto a piece of copper foil (50 μm) and dried at 70 °C for 1 h. The W coating of the Si electrode was conducted using the PVD method (Fig. 1) at Dongwoo Surface Tech Co., Ltd. Ar gas was used as the plasma generator at 100 °C, and W deposition was conducted for 5 min. The deposited W electrode surface was examined by scanning electron microscopy (SEM), transmission electron microscopy (TEM), electron probe X-ray microanalysis (EPMA), and energy dispersive X-ray spectroscopy (EDX).

### Test Cell Procedure

The test cell was assembled with a CR2032-type coin cell in a dry room. The Si anode electrodes were punched out to a size of 14Φ, and the counter electrodes were punched from lithium foil to a size of 16Φ. The measured weight of W nanolayer corresponding to a 14Φ-sized electrode is approximately 0.0001 g. The electrolyte used was 1 M LiPF_6_ with a mixture comprising equal volumes of ethylene carbonate, dimethyl carbonate, and ethylene methyl carbonate (Soulbrain, Republic of Korea). All cells were fabricated in a dry room. The assembled cell was aged for 24 h at 40 °C.

Galvanostatic electrochemical tests were performed using a WBCS 3000 instrument (WonATech Inc., Republic of Korea). Charging and discharging processes were performed between 0 and 1.5 V with specific current rates for each process. After the cycles, surface observations of W-coated and uncoated Si electrodes were conducted. Additionally, impedance tests were performed at frequencies of 10^− 2^ to 10^5^ Hz with an AC amplitude of 5 mV (SOLATRON SI1280B) to compare the coating effect.

## Results and Discussion

**Fig. 2:**
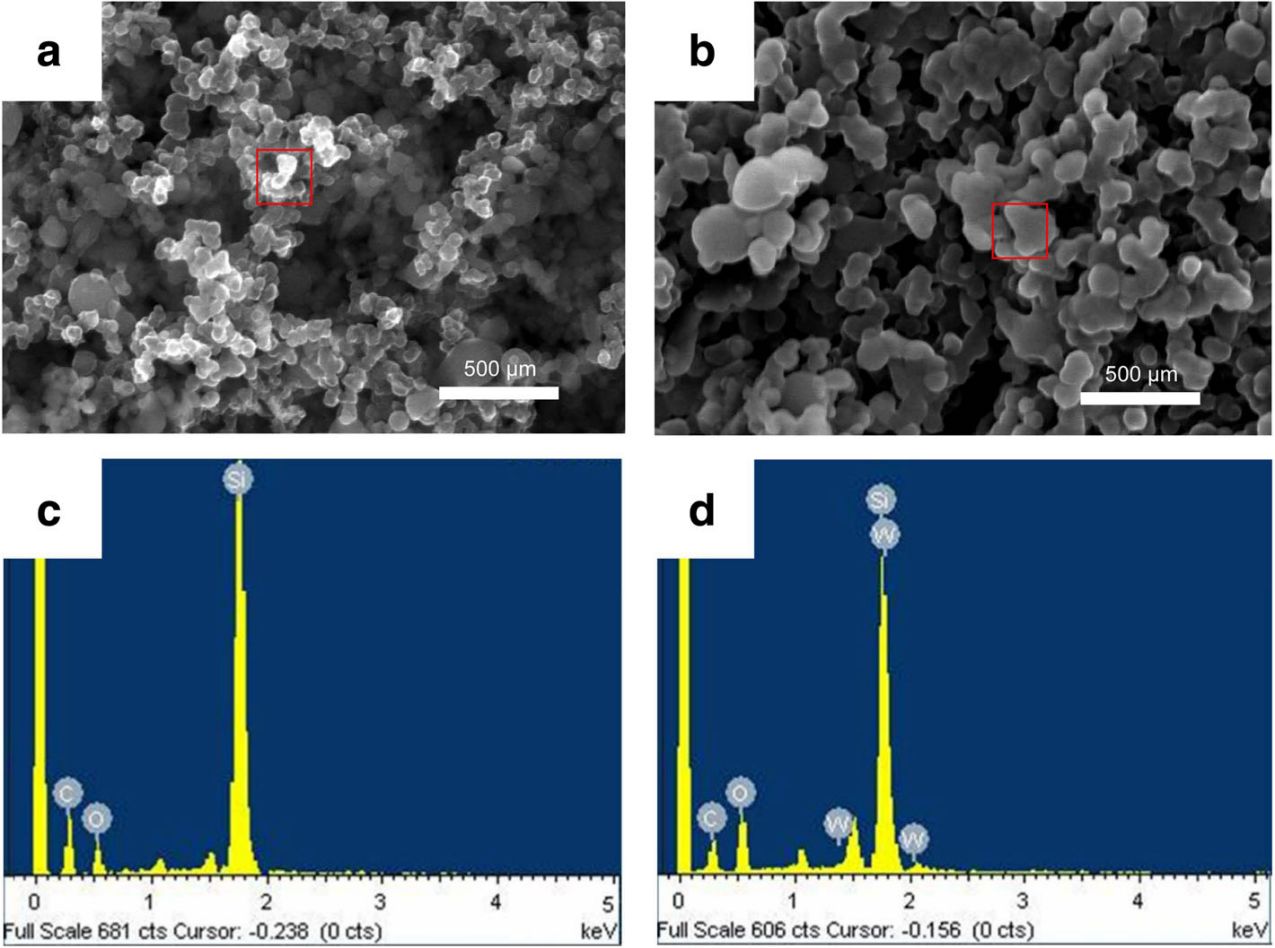
Scanning electron microscopy images and energy dispersive X-ray profile of pristine uncoated **a** and **c** and coated **b** and **d** Si electrode surface

**Fig. 3:**
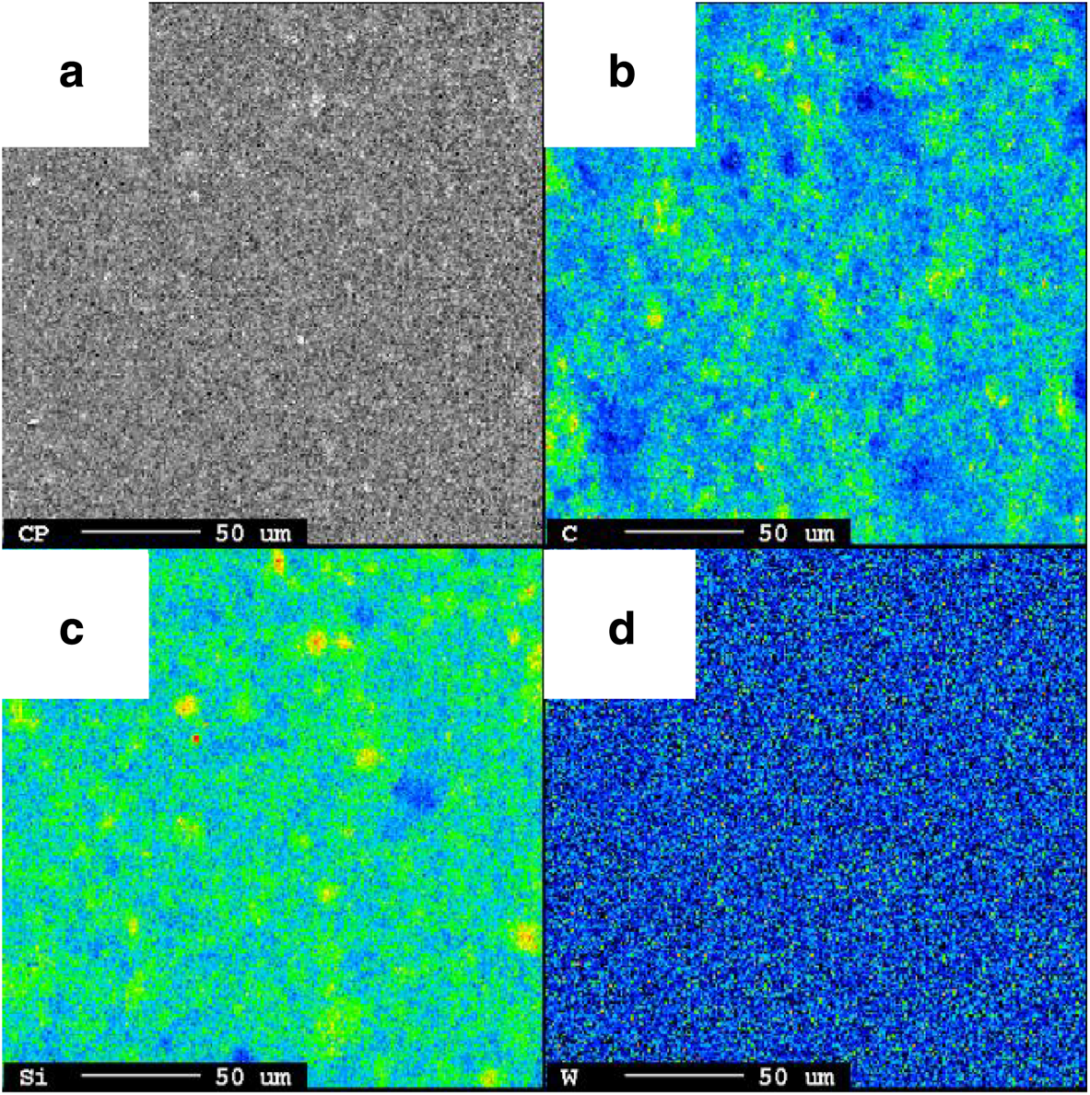
**a** Scanning electron microscopy image and electron probe X-ray microanalysis measurement results of **b** C, **c** Si, and **d** W

Figure 2 shows SEM images of pristine uncoated (a) and W-coated (b) Si electrodes. Because the electrode consisted of Si nanopowder with a size less than 100 nm, the powder retained its original size. However, owing to the physical deposition of W onto the coated electrode, each particle seemed to be covered with a W layer, and the overall size of the particles increased to approximately 100 to 120 nm. EDX analysis of the elements in the red box of the SEM image (Fig. 2b) revealed the presence of W (Fig. 2d). Additionally, EPMA confirmed that the deposited W was uniformly distributed (Fig. 3).

**Fig. 4:**
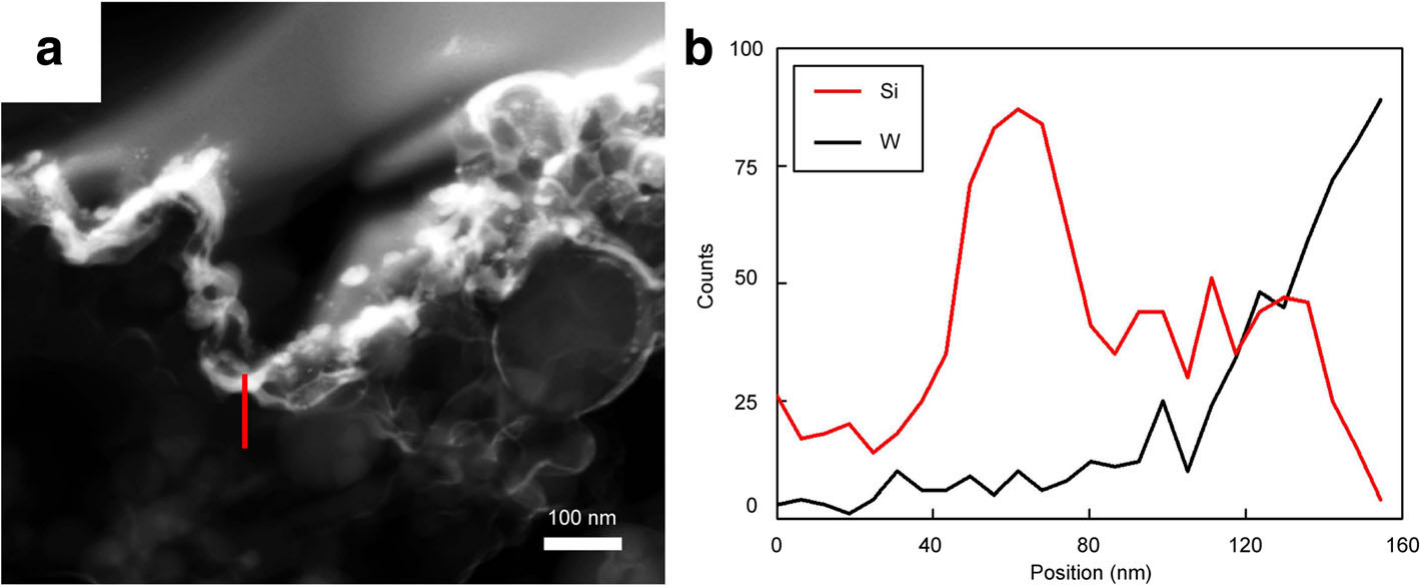
**a** Transmission electron microscopy image and **b** depth profiling of W-coated Si electrode

TEM analysis with depth profiling was conducted to examine the thickness of the W layer. Figure 4 confirms that the W layer (white) deposited onto the Si nanoparticles (black) had a depth of approximately 40 nm. The W layer also covered the gaps between Si powder and other electrode materials. From the above tests, it is apparent that the W layer coated via the PVD method was well formed at the nanometer scale .

**Fig. 5:**
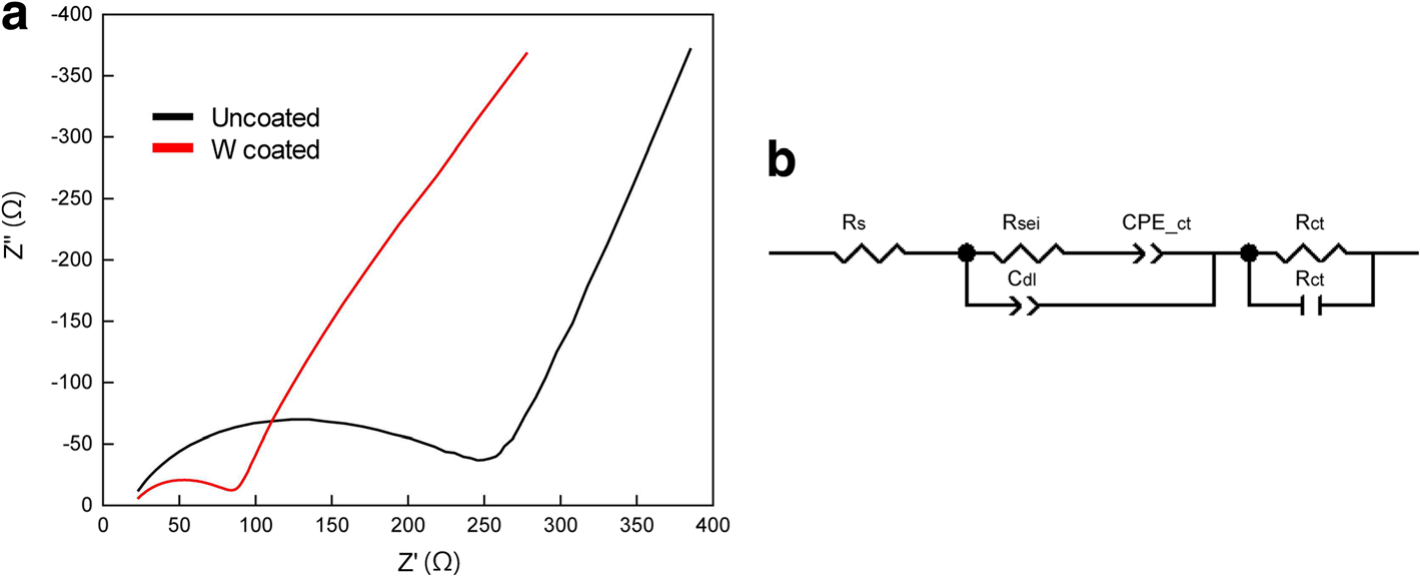
**a** EIS analysis for the uncoated and the W-coated Si electrode before cycles and **b** the equivalent plot

**Fig. 6:**
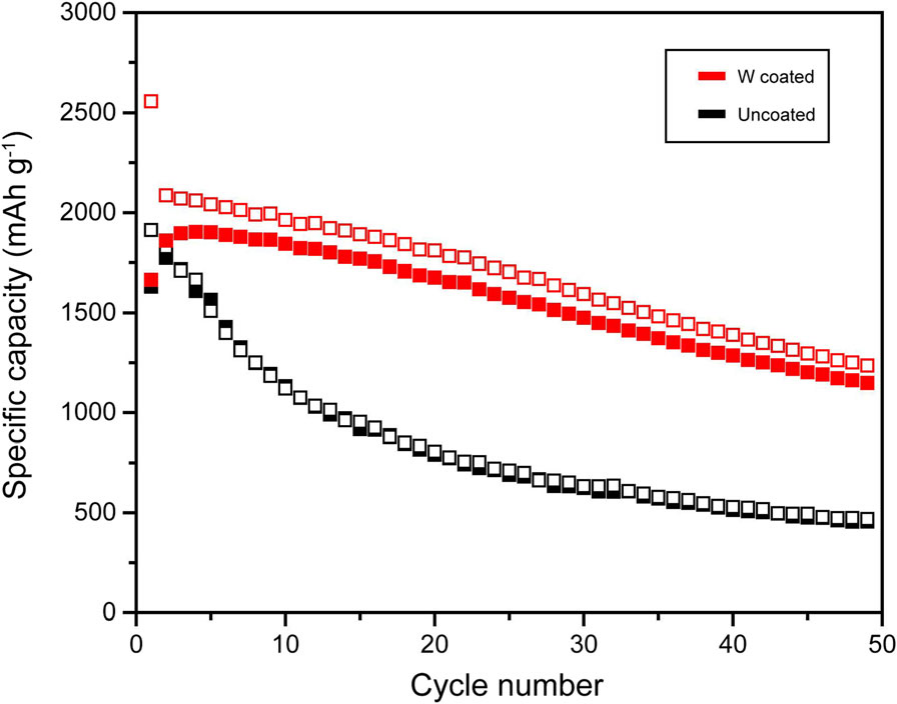
Charge/discharge capacity profiles for uncoated and W-coated Si electrodes at a rate of 0.1 C and cutoff voltage range from 0 to 1.5 V over 50 cycles

**Fig. 7:**
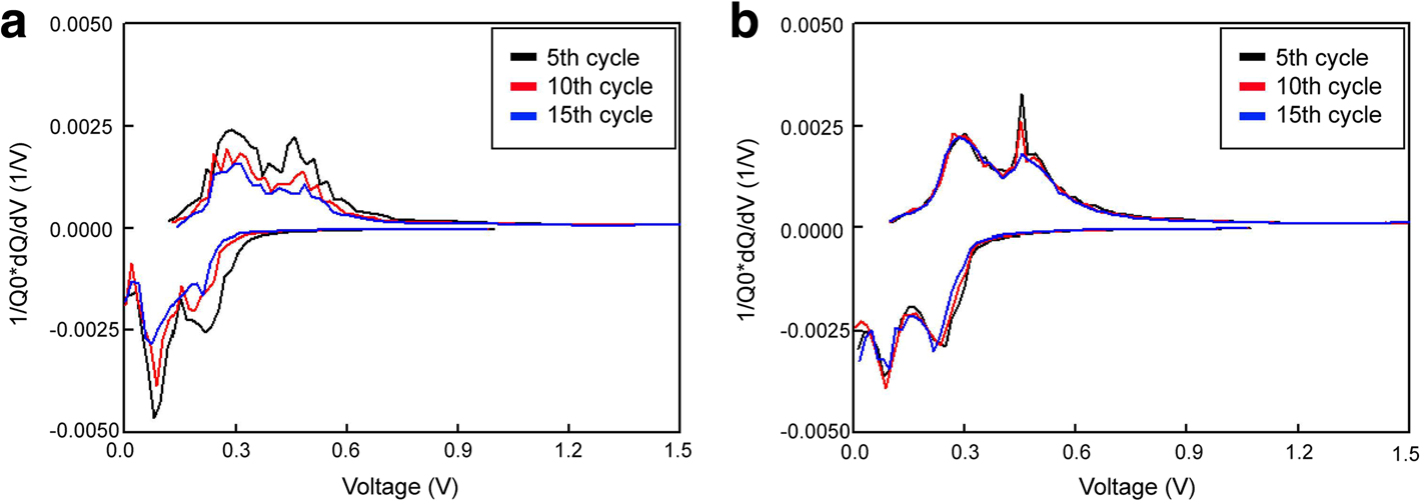
dQ/dV curves for the **a** uncoated and **b** W-coated Si electrode under a rate of 0.1 C with a cutoff voltage range of 0 to 1.5 V (vs. Li/Li+) at the 5th, 10th, and 15th cycles

**Fig. 8:**
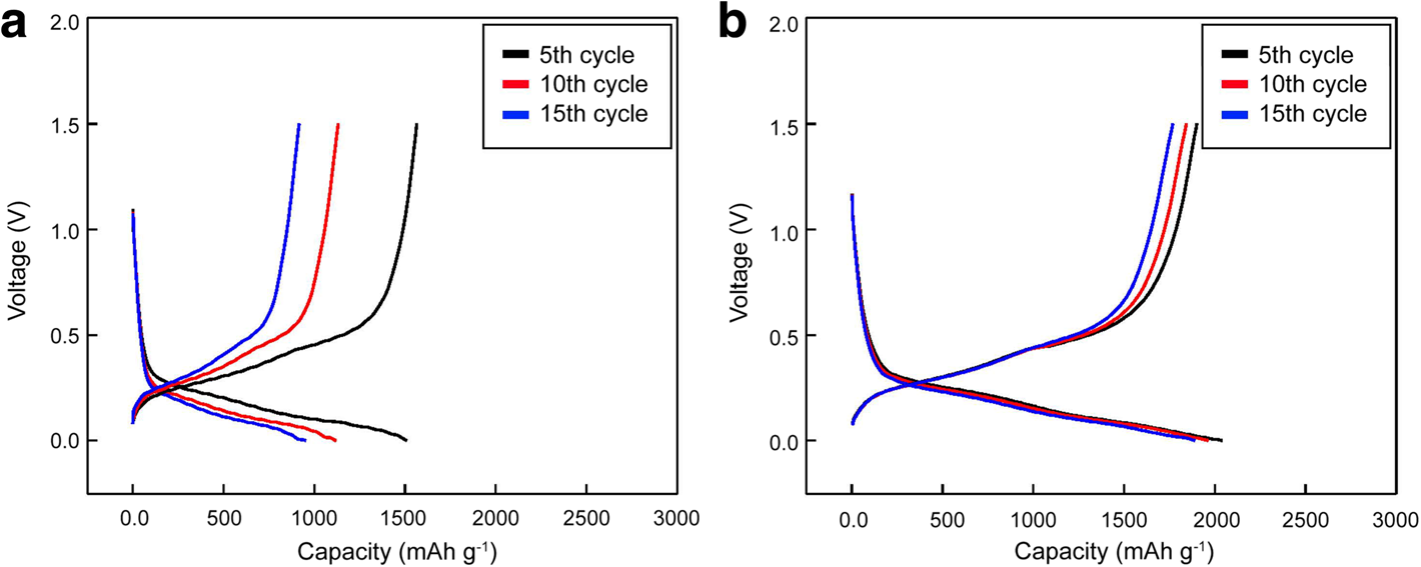
Voltage profiles for the **a** uncoated and **b** W-coated Si electrodes under a rate of 0.1 C with a cutoff voltage range of 0 to 1.5 V (vs. Li/Li+) at the 5th, 10th, and 15th cycles

**Fig. 9:**
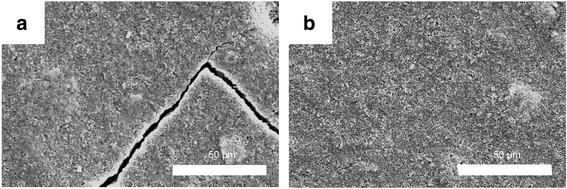
Scanning electron microscopy images of **a** uncoated and **b** W-coated Si electrodes after 10 cycles

An electrochemical impedance spectroscopy (EIS) test was performed for further analysis. Figure 5 shows the impedance results for (a) the uncoated Si and W-coated Si electrodes and (b) the equivalent circuit. The figure shows the equivalent circuit based on the Randles circuit structure, and Table 1 lists the results of impedance fitting. In the equivalent circuit, *R*_s_ indicates the sum of the ohmic resistances of the electrode and electrolyte, and *R*_ct_ and *C*_dl_ represent the charge transfer resistance and double-layer capacitance, respectively. The constant phase element (CPE) is connected to *R*_ct_ in series [25, 26]. *R*_sei_ and *C*_sei_, which are related with the resistance and capacitance of the electrode surface [27], are in parallel.

**Table 1:** Results of impedance analysis fitting data

	Uncoated electrode	W-coated electrode
*R*_s_	20.53	19.37
*R*_sei_	12.36	30.81
*R*_ct_	123.3	24.56
*C*_sei_	7.609 × 10^−4^	7.4236 × 10^−6^

By comparing the initial states, as shown in Fig. 5 and Table 1, the values of *R*_s_ and *R*_ct_ decreased owing to the W coating, whereas *R*_sei_ increased because of the increase in surface resistance. This result indicates that, because of the uniform coating of the W layer, the electrical conductivity was enhanced, which may contribute to increased capacity and stable cyclability. However, the increases in *R*_sei_ and ion diffusion impedance are also observed, implying that the W layer can act as an ion permeability inhibiter.

The specific capacities of the bare and W-coated cells at a rate of 0.1 C over 50 cycles are plotted in Fig. 6. For the first cycle, the charge capacities of the W-coated and uncoated Si electrode cells were 2588 and 1912 mAh g^− 1^, respectively. This may be explained by the high electrical conductivity of W, which allows the Si electrode to receive more Li ions and stimulates faster charge transfer. The discharge capacities of the W-coated Si electrode at the 10th, 20th, and 50th cycles were 1843, 1676, and 1137 mAh g^− 1^, respectively, and the retention ratios of the same cycles were 99.1, 90.1, and 61.1%, respectively. Those values for the uncoated Si electrode were 1132, 790, and 452 mAh g^− 1^ and 63.9, 44.6, and 25.5%, respectively. The coated cell clearly showed improved capabilities. This result is attributable to the W coating, which forms a buffer layer and enhances electrical conductivity. The uncoated Si electrode was exposed to structural destruction, while the W-coated Si electrode was protected by the W nanolayer, preventing the formation of cracks overall and leading to the conservation of the electrode surface. However, the W coating induced irreversible capacity loss during every cycle. Because Li ions must travel through the inactive W layer, which is not an ion-conductive material as discussed in the EIS test, the ion transport during discharging might be sluggish, resulting in irreversibility.

Figure 7 shows the dQ/dV curves of the 5th, 10th, and 15th cycles for both the W-coated and uncoated Si electrodes. The reaction peaks are in the same voltage regions, which imply that the charging and discharging processes occurred with the equivalent reaction [28, 29]. This indicates that the W coating did not influence the morphology of the Si electrode but covered only the surface layer, and it did not act as an active material. As the cycle number increased, the reaction voltage region of the uncoated Si electrode shifted and the polarization increased, whereas the reaction voltage region of the W-coated Si electrode remained relatively constant. This implies that the W coating helps retain chemical stability. This result is also reflected in the voltage profile in Fig. 8, which shows the W-coated electrode preserves its capacity with sustained reaction voltages.

Both the W-coated and uncoated Si electrodes were observed by SEM after 10 cycles (Fig. 9). No cracks were observed on the Si electrode itself, using nanopowder sizes smaller than 100 nm [30]. However, a split occurred during the cycles owing to expansion of the entire electrode. Nevertheless, the W-coated Si electrode remained uncracked, indicating that the atomic deposition by PVD and the intense mechanical strength of W effectively sustained the expansion [19, 20].

## Conclusions

W was coated onto a Si electrode using the PVD procedure to improve the electrochemical performance of the electrode. The coating layer was approximately 40-nm thick and was deposited uniformly. The capacity retention of the W-coated electrode demonstrated enhanced cyclability and was sustained at 61.1% through 50 cycles, whereas the retention of the uncoated electrode was only 25.5%. The surfaces of the two different electrodes were investigated after cycling, and the observations indicated that W acted as a buffer layer. Additionally, the W-coated layer lowered the resistivity of the electrode and enhanced the electrical conductivity of the cell. We hope that this facile nanolayer application through PVD can serve as a reference for future designs of Si-based electrodes.

## Abbreviations

CPE: Constant phase element
EDX: Energy dispersive X-ray spectroscopy
EIS: Electrochemical impedance spectroscopy
EPMA: Electron probe X-ray microanalysis
PVD: Physical vaporization deposition
SEM: Scanning electron microscopy
TEM: Transmission electron microscopy
